# Supplementary material for: Evidence for Beneficial Physiological Responses of the Land Snail Cornu aspersum to Probiotics’ (Lactobacillus plantarum) Dietary Intervention
Source: Animals (Basel). 2024 Mar 11;14(6):857. doi: 10.3390/ani14060857 (PMC10967277; doi:10.3390/ani14060857)

Evidence for Beneficial Physiological Responses of the Land Snail *Cornu aspersum* to Probiotics' (*Lactobacillus plantarum*) Dietary Intervention

Efstratios Efstratiou [1], Konstantinos Feidantsis [2], Vasiliki Makri [1,3], Alexandra Staikou [3,*] and Ioannis A. Gi-antsis [1,*]

1 Faculty of Agricultural Sciences, University of Western Macedonia, GR-53100 Florina, Greece; str.efstratiou@gmail.com (E.E.); makrivasil@bio.auth.gr (V.M.)

2 Department of Fisheries and Aquaculture, University of Patras, GR-26504 Mesolonghi, Greece; kfeidant@upatras.gr

3 Department of Zoology, School of Biology, Aristotle University of Thessaloniki, GR-54124 Thessaloniki, Greece

* Correspondence: astaikou@bio.auth.gr (A.S.); igiantsis@uowm.gr (I.A.G.)

Figure S1: The complete original immunoblots shown in Figures 4 and 5 are presented in order below. The individual parts comprising Figures 4 and 5 are specified using black boxes.

Cropped blots in main paper

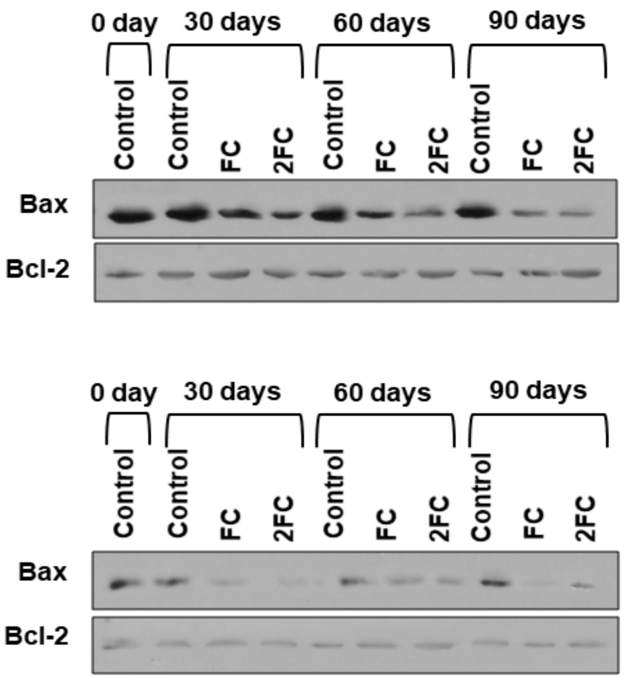

Original blots

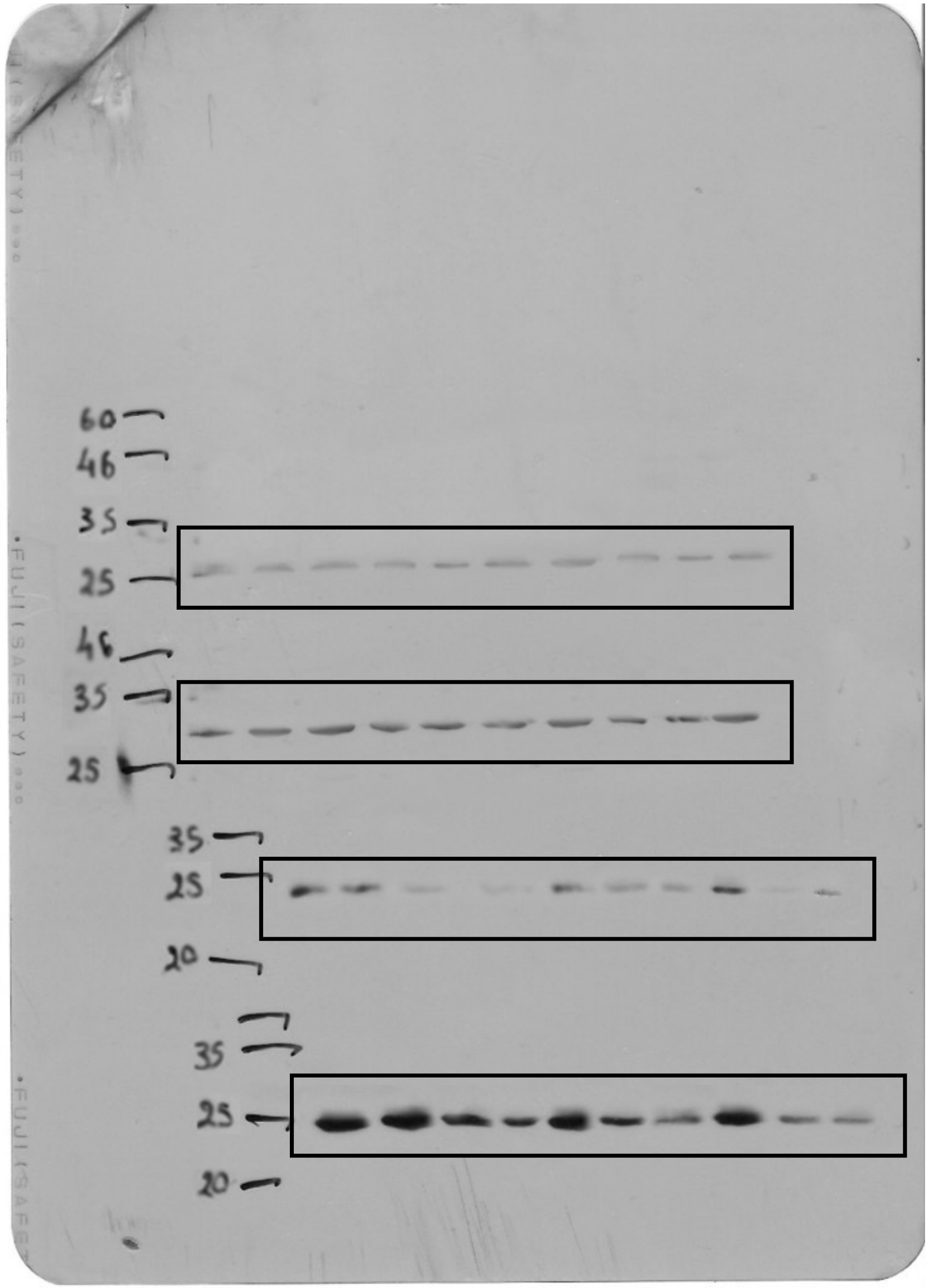

Figure S2: The complete original immunoblots shown in Figure 6 are presented in order below. The individual parts comprising Figure 6 are specified using black boxes.

Original blots

Cropped blots in main paper

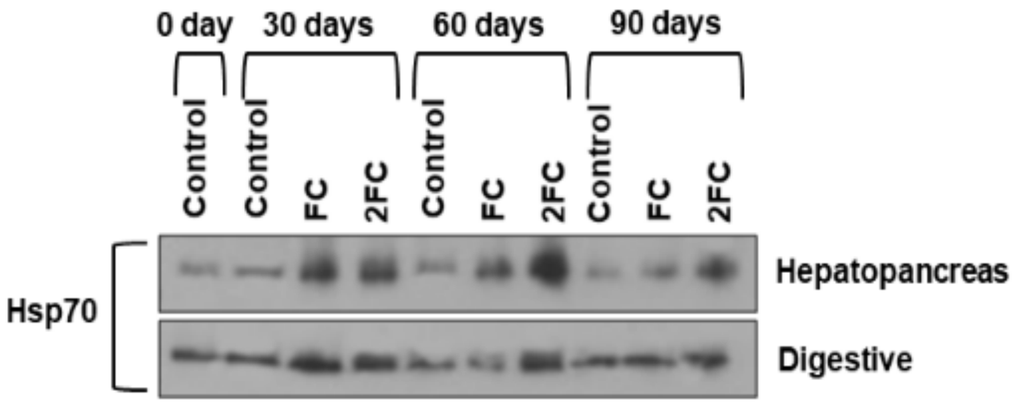

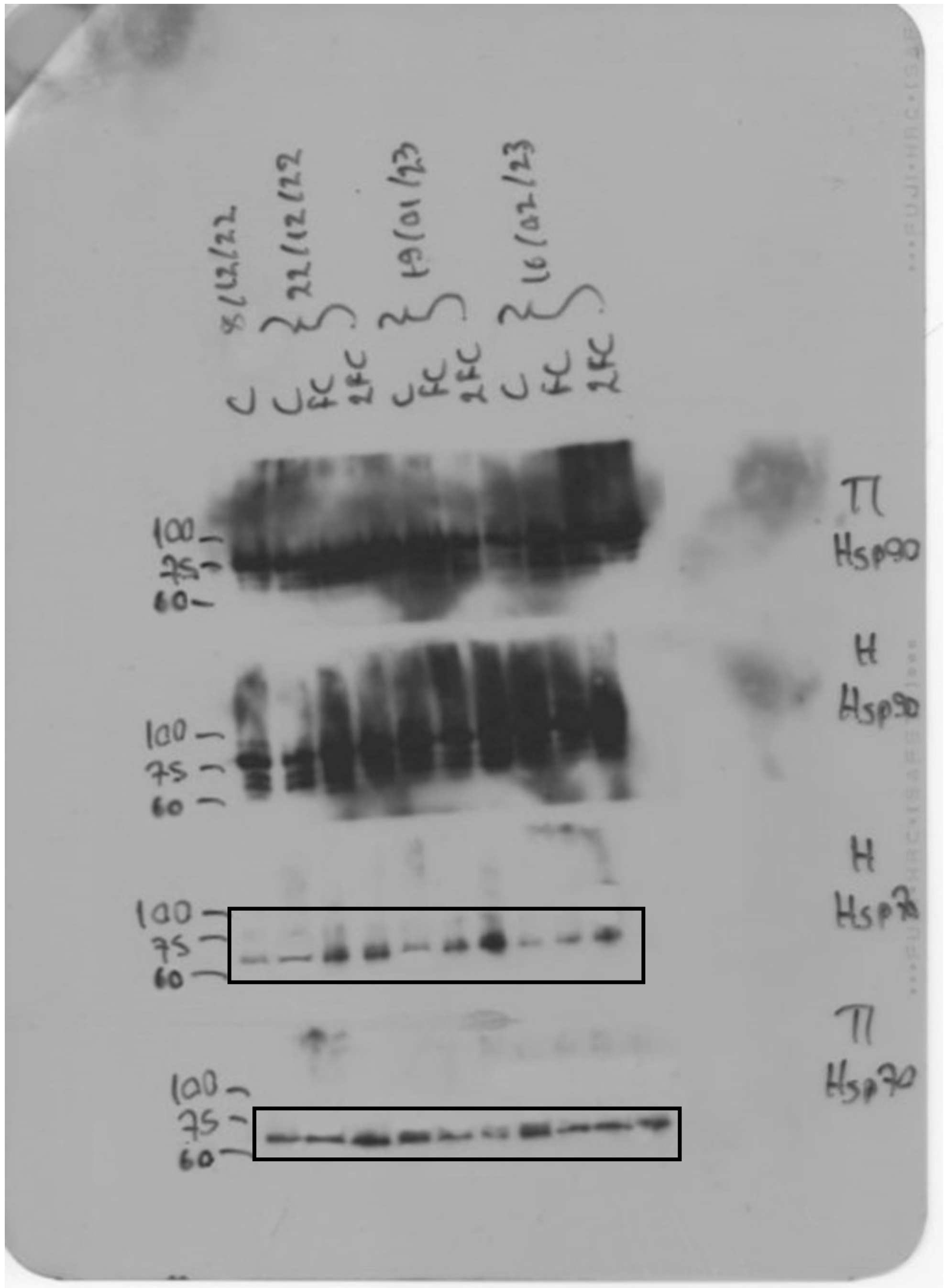

Figure S3: The complete original immunoblots shown in Figure 7 are presented in order below. The individual parts comprising Figure 7 are specified using black boxes.

Cropped blots in main paper

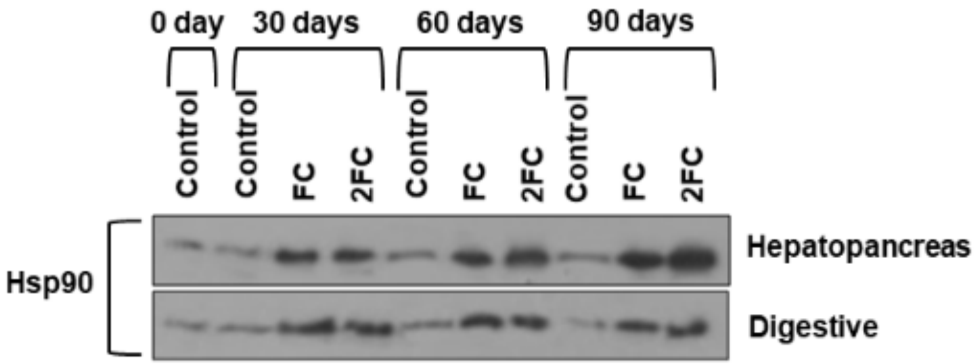

Original blots

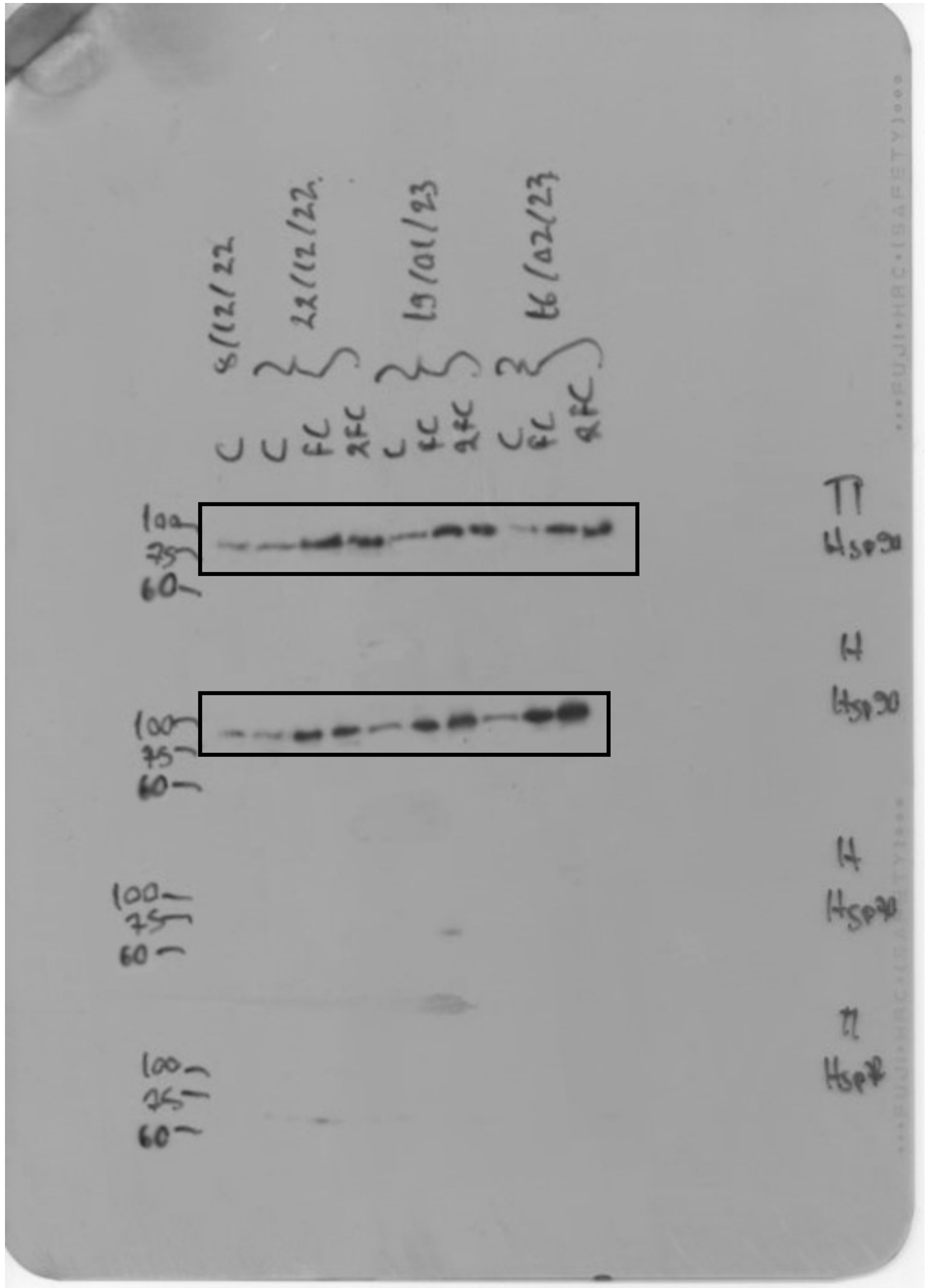

Figure S4: The complete original immunoblots shown in Figures 4-7 are presented in order below. The individual parts comprising Figures 4-7 are specified using black boxes.

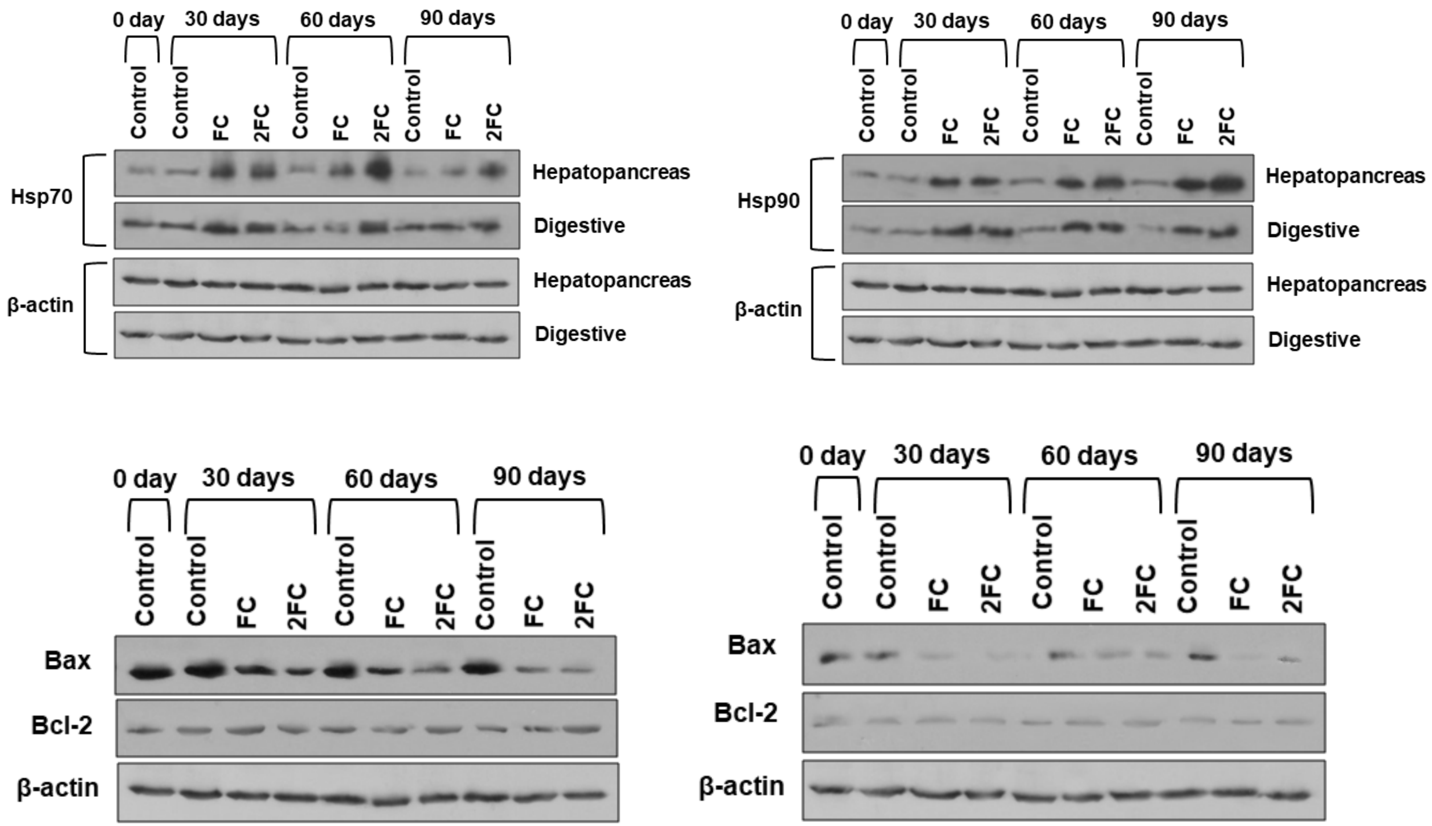

Original blots

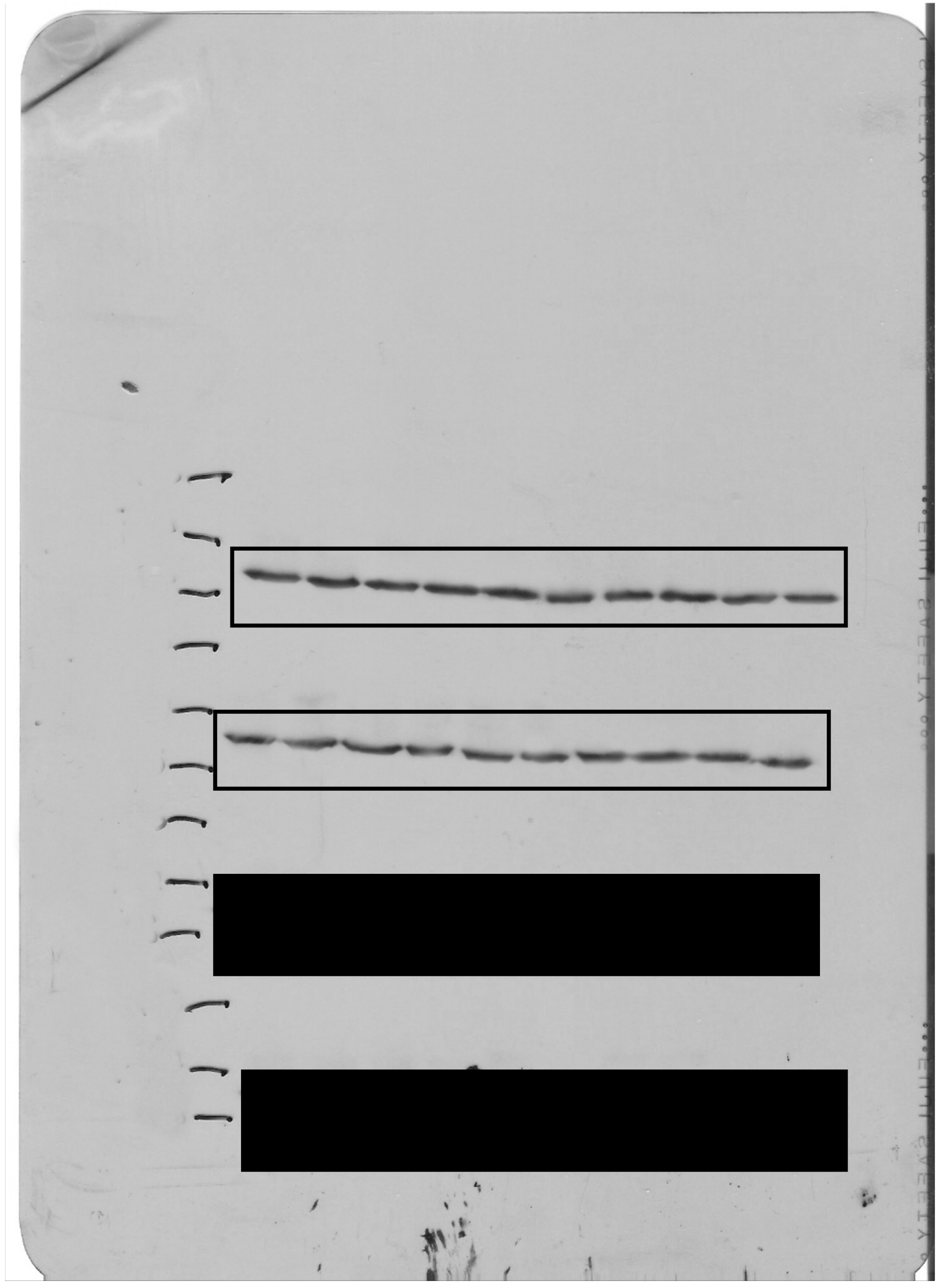

Supplement: Supplementary file 1 [file animals-14-00857-s001.zip › animals-2815111-supplementary.pdf]
